# Workplace Incivility and Job Satisfaction: Mediation of Subjective Well-Being and Moderation of Forgiveness Climate in Health Care Sector

**DOI:** 10.3390/ejihpe11040082

**Published:** 2021-09-22

**Authors:** Muhammad Safdar Khan, Natasha Saman Elahi, Ghulam Abid

**Affiliations:** 1 School of Business Administration, National College of Business Administration & Economics, Lahore 54660, Pakistan; drsaldera@yahoo.com; 2 Department of Business Studies, Kinnaird College for Women, Lahore 54660, Pakistan; ghulam.abid@kinnaird.edu.pk

**Keywords:** workplace incivility, job satisfaction, subjective well-being, forgiveness climate

## Abstract

Our study investigates the role of subjective well-being and forgiveness climate between workplace incivility and job satisfaction. Drawing on conservation of resource theory, we proposed a model in which workplace incivility is associated with job satisfaction through subjective wellbeing, and forgiveness climate moderates this association. Data was collected through a survey method from 672 nurses and doctors in the health care sector at two different times. Respondents completed workplace incivility and subjective well-being scale at Time 1, and a forgiveness climate and job satisfaction scale at time 2. Findings through PROCESS Macros (Model 5) show that workplace incivility has a negative influence on job satisfaction and subjective well-being. Subjective well-being plays a mediating role in the negative effect of workplace incivility on job satisfaction. Moreover, forgiveness climate moderates the relationship between workplace incivility and job satisfaction. The implications for practice and research are discussed.

## 1. Introduction

Nurses play an essential function in the health sector. They work 24 h a day and seven days a week to look after patients and communicate with them. They must provide excellent treatment, and they are a vital source of hope for patients seeking the best possible healthcare services. Unsurprisingly, nurses have experienced uncivil behaviors in the workplace, which may come from patients, doctors, supervisors, or other nurses [1]. Ignoring, omission, humiliating, aggressive looks, eye-rolling, interruptions, gabbing, insulting, and disrespecting are examples of uncivil behavior. These kinds of behavior lead directly to workplace incivility that, as low-intensity deviant behavior with an unknown intent to damage the receiver, violates organizational norms of mutual respect. Uncivil behavior includes rudeness and discourtesy, and a lack of consideration for others [2]. The most prominent antisocial behavior people encounter in the work environment is workplace incivility. For example, [3] demonstrated that as many as 98 percent of US workers have dealt with uncivil behavior at work. The authors of [4] found that 76 percent of 303 American nurses surveyed had encountered incivility at some point during their careers. A survey of 117 inexperienced Canadian nurses discovered that 90.4 percent had experienced some kind of co-worker incivility during their early careers [5]. Since workplace incivility has an undesirable impact on the workplace and patient care, its influence in hospital environments is enhanced. For example, nurses have stated that workplace incivility causes emotional distress and distractions in the workplace. It increases the chances of making mistakes in the patient’s care and puts patients at higher risk. Workplace incivility lowered the quality of nurse’s treatment they rendered [6], their motivation in the workplace [7], and enhanced their posttraumatic stress disorder [8]. Incivility is related to negative results, which may put financial pressure on healthcare organizations and advance the nurses’ turnover [9]. Nurses who have experienced workplace incivility had a 92 percent greater risk of enduring illness absence [10].

In the health sector, several harmful outcomes had determined less about the underlying mechanism by which workplace incivility has a detrimental impact on job-related consequences. There is also a scarcity of information about job resources (forgiveness climate) that could help to minimize the negative influence of workplace incivility on job-related outcomes [11]. It is essential to define the mechanism as they could act as potential suspects for interventions from the viewpoint of both researchers and practitioners. Likewise, numerous studies have observed that workplace incivility adversely influences attitudinal outcomes. Fewer have looked at its relationship with job satisfaction. It is especially crucial to identify workplace predictors that reduced job satisfaction. This is because job satisfaction is associated with happiness and job performance [12], organizational commitment [13], organizational citizenship behavior [14], and lower turnover intention [15]. It also retains and attracts well-qualified personnel in the health sector [16]. On the other hand, we look at subjective well-being as one pathway that could describe the connection between workplace incivility and job satisfaction in the current study. Subjective well-being is defined by [17] as perceptions of people about their existence or their perspective on their life experience. There is a mountain of research demonstrating that subjective well-being envisages a variety of consequences [18] incorporating higher psychological functioning and health longevity [19].

Our study also anticipates that a forgiveness climate may reduce the detrimental impact of workplace incivility on job satisfaction. Forgiveness climate is an environmental abstraction focused on the daily experiences of employees [20]. Forgiveness climate reduces punitive intent in response to ethical misconduct [21]. It enhances members’ positive emotions, such as empathy, sympathy, or love, that improve their innovative behavior. It increases positive thinking and subjective happiness [22]. The role of a forgiveness climate has not been explored in reducing workplace incivility and job satisfaction association. Prior studies examined the role of forgiveness climate between psychological contract breach and emotional exhaustion [23]. In sum, the present study addresses such a particular void in the literature in the health care setting. Initially, we use the conservation of resources (COR) theory [24,25] to anticipate and illustrate that resource-draining workplace incivility can lead to a decrease in job satisfaction due to a lack of subjective well-being [24,25].When workers’ resource reserves have diminished due to negative work conditions, such as incivility, they do not show positive behavior and devote all of their energy resources to coping with their preoccupations with organizational performance [24]. Few studies have identified the mechanism by which workplace incivility is related to negative work-related outcomes such as lower job satisfaction. This research aims to address this limitation by introducing subjective well-being as the mechanism that connects workplace incivility to job satisfaction. Furthermore, the forgiveness climate has been described as a moderator that may reduce the destructive impact on job satisfaction. Lastly, our research focuses on Pakistan, a little-studied non-Western country that should be extremely important to the evaluated theoretical model since this country is accompanied by increased levels of risk avoidance. Individuals with a high cultural connection to their country may be furious by poor working conditions that introduce instability to their organizational performance, and workplace incivility is a tenacious challenge in numerous Pakistani working environments [26]. The current study is being carried out in the Asian health care industry in order to address the following research questions. Does workplace incivility indirectly influence job satisfaction through subjective wellbeing? Does forgiveness climate moderate workplace incivility and job satisfaction relationship?

## 2. Literature Review and Hypotheses Development

### 2.1. Theoretical Perspective

We used COR [24] to establish a theoretical justification for why workplace incivility can influence job satisfaction. This theory stated that people endeavor to preserve, secure, and generate resources. Resources are personal characteristics, objects, circumstances, or energies that individuals’ value or that serve as a basis for acquiring these resources. Stressors in the working environment reduced these resources. Individuals encounter stress if these resources vanish, challenge, or are not appropriately replenished [27]. Therefore, to save resources, people tend to prevent stress factors. This theory states that social relationships are a form of specific resource that may deplete or supply the respective resources [24]. In this view, workplace incivility is a social stressor that can endanger employee social relationships and decrease employee affective and cognitive resources. Individuals who face more uncivil behavior are more likely to have negative feelings and have less social and emotional energy [28]. Employees must devote time and attention to dealing with these uncomfortable experiences, depleting their scarce resources for work-related activities. In line with the theory, we speculated in this study that losses of emotional and cognitive resources caused by workplace incivility may result in lower subjective well-being (SWB) and job satisfaction.

### 2.2. Workplace Incivility, Subjective Well-Being, and Job Satisfaction

Workplace incivility is insensitive and rude behavior that displays a lack of respect for others [2]. Workplace incivility is unrestrained, exists almost in every workplace [29], and its ratio is increasing in the workplace [30]. Supervisors, colleagues, and clients can instigate incivility in organizations. Common examples of uncivil behavior are responding to phone calls in an impolite way, speaking abusive language about another worker, and sending a discourteous email to a collaborator [29]. It also includes obtaining the privilege of the people’s knowledge, idea, and work, spreading rumors about colleagues, not supporting subordinates, refusing colleagues’ requests, yelling at peers, and underestimating peers’ different views. Over the past 15 years, the literature has empirically shown that incivility in the workplace is associated with detrimental consequences for its targets [31]. The organizational and individuals cost of this low-intensity behavior are a sense of job insecurity [32], work-family conflict, lower innovative work behavior [33], higher turnover [30], burnout, lower helpfulness, task performance, creativity, and perceived insider status [34]. Workplace incivility indirectly influences organizational citizenship behavior through burnout. Moreover, the positive impact of low-intensity action (workplace incivility) on employee burnout is high with higher affective commitment, indicating that low-intensity behavior may be more damaging to individuals when they have higher affective commitment [35]. [11] observed that incivility is negatively associated with symptoms of insomnia and negative job rumination. Nurses and doctors in the hospitals try to acquire resources (i.e., prosocial motivation and self-efficacy) that could support their extra-role behavior (organizational citizenship behavior) and social connections to cope with the difficulties of the job environment [36]. Workplace incivility reduced nurses compassion competence [37]. It increased counterproductive behavior and negative emotions. The experience of uncivil behavior creates dissatisfaction among employees [38]. Workplace incivility led to a loss of resources in the form of burnout, which anticipated workplace withdrawal and job satisfaction [39]. Job satisfaction is how people feel about their work and various facets of their jobs [40]. Therefore, in line with theory:

**Hypothesis 1** **(H1).**  *Workplace incivility is negatively associated with job satisfaction.*

Workplace incivility negatively influences the employee well-being, job attitudes, and health [31], stress [32], anxiety, depression, vengeance against the organization [41], and higher psychological distress. The authors of [42] found that workplace incivility adversely influences physical and mental well-being. In line with COR theory, individuals experience stress such as incivility while working, then these resources are lost, destroyed, or not sufficiently refilled [27]. Incivility in the workplace is a relational stressor [43]. Therefore, it is a disruption to healthy working relationships [2]. Good relationships are essential for the career development of people. Incivility can disrupt the social relationships of employees and ultimately reduce other forms of resources in the workplace [27]. Incivility targets can see the uncivil experience as a challenge to their social self-esteem or social status in the workplace economic stability, perceived self-worth, or perceived job security [44]. Incivility can drain the personal resources of the targets if they conduct uncivil encounters. Previous research has validated that incivility in the workplace compromised personal resources and negatively connected [45] to subjective well-being. Additionally, the experience of low-intensity behavior is emotionally and cognitively exhausting [43]. In dealing with these uncomfortable experiences, workers devote energy and time, bringing down the scarce resources available for job-related duties. The authors [46] stated that employees who experience uncivil behavior do not have additional resources to invest, because they have already used those resources at work for regulating and processing their responses to low-intensity experiences. For that reason, they would not experience subjective well-being. It refers to how individuals assess their own lives, contain affective evaluation (positive and negative emotional feelings), and cognitive assessment (life satisfaction) [17,47]. It is higher mental health, behavioral styles of self-improvement, and interpersonal connection. Prior studies have determined that factors can improve the subjective well-being of employees i.e., i.e., thriving, civility, fairness perception [48], flourishing, energy [49], ethical leadership, voice behavior [50], and work/life balance [51], while the influence of workplace incivility on employee subjective wellbeing was not explored. So, we posit:

**Hypothesis 2** **(H2).**  *Workplace incivility is negatively associated with subjective wellbeing.*

Job satisfaction is a pleasant or positive emotional state that results from the appraisal and experience of one’s job [52]. Satisfied employees contribute to organizational success and performance [53]. The authors of [54] found that job satisfaction is associated with leadership and performance, motivation, attitude, and voice behavior [55]. Therefore, prior studies found factors that can improve the job satisfaction of employees in the workplace. In those studies, [56] pointed that supervisor ethical leadership increases job satisfaction through moral identity and moral awareness. In turn, [57] found that supervisor cooperation, career growth, and work atmosphere enhance job satisfaction. Likewise, employees are more satisfied with their job when they get the supervisor’s humanity [58]. Likewise, perceived organizational support, psychological empowerment [59], and thriving at work [60] are predictors of job satisfaction. Thus, we proposed that subjective well-being would enhance employees’ job satisfaction. Individuals who have a positive evaluation of their lives are more satisfied with life and have positive feelings. Happy employees are more efficient in several life domains, and have good health and social relationships. They are highly creative and involved in prosocial actions. Further, they have coping and problem-solving skills. In addition, employees with higher subjective well-being do not leave their job, perform better [49], and are more involved in the extra-role activities as compared to those employees who have low satisfaction [18]. In the work satisfaction model, [61] theorized that affective traits predict work satisfaction. [62] empirically tested the work satisfaction model of [61,63] and showed that individuals are more likely to have a positive attitude towards their work (i.e., work satisfaction) when they have a higher level of positive affect. Other studies have also examined the relationship between positive affect (one element of subjective well-being) and work satisfaction. For example, a meta-analysis indicated positive and negative effects are correlated with job satisfaction. In another meta-analysis reported a correlation between positive affect and job satisfaction [61,62]. These studies suggested that individuals who are more likely to be satisfied with their jobs are those who generally experience positive emotions. Studies showed that these employees are more productive, are highly engaged, have proactive behavior, and are more satisfied with their job. Further, they are more audacious, feel happiness at the job, are more conscious about their personal growth, and perform all their tasks and job duties effectively. They are mentally and physically fit [64].

**Hypothesis 3** **(H3).**  *Subjective well-being is positively associated with job satisfaction.*

Aligned with COR theory and the above-mentioned hypothetical relationships, we assume that workplace incivility lowers workers’ subjective well-being, resulting in lower job satisfaction. Employees with lower SWB feel stress, emotional exhaustion, a physical ailment that results in burnout, absenteeism, and turnover [18]. Individuals strive to defend and conserve their ‘resources’ (any attributes or conditions desired by the person) whenever necessary. This situation is particularly evident in work settings. However, individuals face the dilemma of replenishing their resources whenever confronted with a negative situation (workplace incivility) in which resources are exhausted. We suggested in this study that workplace incivility is a psychological stressor that can challenge workers’ emotional and cognitive resources, leading to lower subjective well-being that can in turn lead to lower job satisfaction. Subsequently, we posit that:

**Hypothesis 4** **(H4).**  *Workplace incivility indirectly influences employee job satisfaction through subjective well-being.*

### 2.3. Moderating Role of Forgiveness Climate between Workplace Incivility and Job Satisfaction

The authors of [65] stated that the forgiveness climate emphasizes employee perceptions of behavior that ascend every day and are strengthened by the organization. The forgiveness climate implies that when employees face a conflict or offense from another organizational member, they display tolerance and kindness predicted from the organization [66]. It entails avoiding accusations, hatred, and anger towards the individuals who commit a mistake by taking a tolerant approach to errors in general [67]. The climate of forgiveness affects the organizational member’s actions by providing appropriate social signals in response to workplace offenses. Researchers noted that job resources such as organizational, supervisor, and co-worker support (supportive work environment), team climate, and psychological safety promote positive outcomes for employees [68]. For example, higher commitment with organization, job performance, organizational citizenship behavior, lower turnover intention [69], well-being, and work engagement [70]. Job resources are psychological, physical, organizational, or social facets of the job that minimize work demands; are functional to achieve job objectives; and promote personal learning, growth, and development [25]. Employees’ personal development and their readiness to providing their efforts towards obligations are associated with work. These are improved by such resources and eventually positively influence their job and individuals’ outcomes [69,70]. In organizations where the atmosphere of forgiveness is predominant, people prefer to stop grudging as much as possible, and restrain from blaming one another when tolerating errors and accepting mistakes [71]. An earlier study finds that if workers can respond to tolerable harms, they can change their emotions and restore damaged interpersonal relationships. Forgiveness climate is considered a contextual factor that really can help employees to establish positive interpersonal relationships. Employees can feel support from the organization and reward their tolerance and mercy in the higher forgiving climate. Forgiveness in the workplace is associated with psychological and physiological recovery [72]. Forgiveness enhances people’s happiness, and the happiest people are likely to forgive rather than less happy people [73]. Forgiveness can directly provide a protective factor against depression in adolescents. Forgiveness potentially offers a preventive mechanism for adolescents toward depression by helping them to manage and control emotions, thus improving emotional health. Forgiveness fosters positive working relationships and promotes a pleasant and stable workplace. Forgiveness for mistakes is often likely to minimize depression and stress levels [71]. A forgiving atmosphere that deters blame and retribution prohibits workers from wasting their energy/energies to cope with negative feelings such as remorse, embarrassment, and fear they could utilize all their resources for efficient service retrieval. Further, [69,71] found that forgiveness climate enhances job satisfaction. For that reason, we proposed that forgiveness climate as a job resource weakens the adverse influence of incivility on job performance. Thus, we anticipate:

**Hypothesis 5** **(H5).**  *Forgiveness climate moderates the relationship between workplace incivility and job satisfaction.*

**Figure 1:**
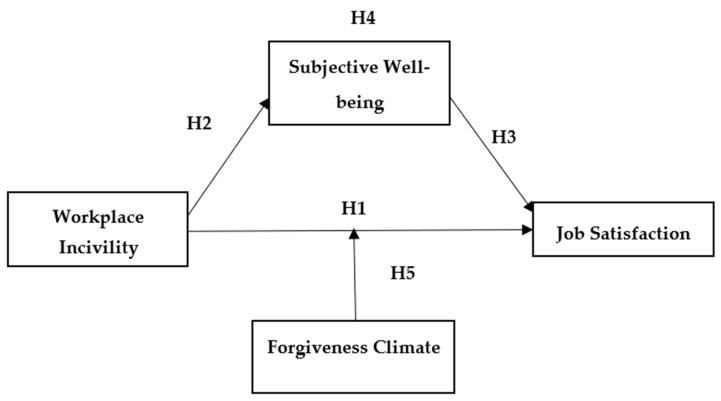
Theoretical Model.

All relationships are summarized in Figure 1.

## 3. Methods

In this study, data were gathered from different hospitals by applying the non-probability sampling technique. Doctors and nurses were considered the most suitable responders. The purpose of the study was to know more about the people who treated patients in hospitals. The data were collected from the respondents by a self-administered questionnaire employing a time-lagged structure. Therefore, data on workplace incivility and subjective well-being was obtained at Time 1. At Time 2, data on forgiveness climate and job satisfaction was collected. A total of 760 questionnaires were disseminated in various hospitals to obtain the desired response and achieve our goal. Respondents were asked to give a response about study variables according to their personal opinions. A total of 672 out of 760 valid responses were collected from respondents with an 88.1 percent response rate.

### Study Instruments

Workplace incivility was measured with a seven-item scale from [32]. One example item is “Paid little attention to your statement or showed little interest in your opinion?”. This measure was operationalized on a five-point Likert scale ranging from 1 (never) to 5 (frequently). Subjective well-being was measured by the Satisfaction with Life Scale of [47] An example item is “The conditions of my life are excellent”. This scale was measured using a five-point Likert scale varying from 1 (completely false) to 5 (completely true) and consisted of three items. Forgiveness climate was been measured with a three-item scale of [71]. One sample item is “We forgive each other’s errors/mistakes/offenses”. We measured job satisfaction with the three-item scale of [74]. One sample item is “In general, I like working here”. For forgiveness climate and job satisfaction, both scales were anchored on a five-point Likert scale varying from 1 (strongly disagree) to 5 (strongly agree).

## 4. Results

### 4.1. Confirmatory Factor Analysis

The hypothesized model including all four constructs (workplace incivility, subjective well-being, job satisfaction, and forgiveness climate) generated a good fit to data, i.e., CMIN/DF (1.89), CFI (0.97), IFI (0.97), NFI (0.95), RMSEA (0.03), and TLI (0.97), in comparison to the single-factor model, which combined all variables into one variable. This alternate model does not yield the best fit to data as CMIN/DF (22.13), CFI (0.44), IFI (0.44), NFI (0.37), RMSEA (0.17), and TLI (0.43) (Table 1) [75].

### 4.2. Construct Validity

The composite reliability (CR) and average variance extracted (AVE) values of all constructs surpassed the satisfactory limit >0.7 and >0.5, demonstrating convergent validity. Discriminant validity demonstrated by the square root of the AVE value of all constructs was greater than the intra-construct correlation [76] (see Table 2).

### 4.3. Reliability and Correlation Coefficient

The correlation of workplace incivility was found to be negative and statistically significant with subjective well-being (r = −0.13 **), job satisfaction (r = −0.17 **), and forgiveness climate (r = −0.11 **). Next, the correlation of subjective wellbeing was statistically significant and positive with job satisfaction (r = 0.26 **) and forgiveness climate (r = 0.16 **). The correlation of job satisfaction was found to be positive and significant with forgiveness climate (r = 0.35 **) (Table 3).

### 4.4. Direct, Indirect, and Conditional Effects

This study used Process Macros in SPSS 24 (Model 5, 5000 bootstrapping, 95 CI) to inspect the directional dependency of the study variables. The direct, indirect, and conditional effects of study variables were presented in Table 4. The outcome revealed a significant and negative influence of workplace incivility on job satisfaction (β = −0.46, *p* < 0.001, supporting H1) and subjective well-being (β = −0.11, *p* < 0.001, supporting H2). The results also showed a positive and significant effect of subjective well-being on job satisfaction (β = 0.14, *p* < 0.001, supporting H3). The outcome revealed a significant and negative influence of forgiveness climate on employees’ subjective wellbeing. Next, an indirect effect of workplace incivility on job satisfaction through subjective wellbeing was negative and significant (β = −0.016, LB = −032, UB = −006, supporting H4). Furthermore, the interaction effect of workplace incivility and forgiveness climate (WPI×FC) on job satisfaction was found to be significant (β = 0.10, *p* < 0.001, supporting H5).

## 5. Discussion

Based on COR theory [24,25], the present research studied the indirect effect of workplace incivility on job satisfaction through subjective well-being. In addition, it examined whether the relationship between workplace incivility and job satisfaction was conditional on forgiveness climate in the Asian health sector [26]. General, the study findings support the hypothesized model. In particular, workplace incivility negatively influenced job satisfaction. These findings are consistent with prior results that found that uncivil behavior creates dissatisfaction among employees [38,39]. Our study found that workplace incivility was negatively associated with subjective well-being. These results have favored the results of [31,42] that found that workplace incivility influences employees’ well-being. In turn, it influenced job satisfaction. These results supported the prior studies that demonstrated that subjective well-being creates beneficial outcomes [18,19,64] i.e., job satisfaction [61,62,63]. This evidence advocates a mediated relationship in which subjective well-being was the underlying mechanism that describes the association between workplace incivility and lower job satisfaction. Additionally, in line with study [23], our study studied forgiveness climate as potential moderators in the workplace incivility and job satisfaction association. Forgiveness climate at workplace helps in minimizing the negative effect (the overall effect on job satisfaction is positive) of workplace incivility. In general, these effects were higher for those nurses and doctors who were involved in the forgiveness climate. These results lead to several organizational interventions that could reduce the negative impact of workplace incivility while also improving employee subjective well-being and job satisfaction, which we address in the subsequent paragraphs.

### 5.1. Theoretical and Managerial Implications

Our study contributed to literature in the following ways. Firstly, we proposed subjective well-being as an underlying factor for explaining the link between workplace incivility and job satisfaction. Findings support the indirect impact following the COR theory [24], adding to the expanding body of research supporting this theory. The study findings are extended to the occupational health psychology research [37], demonstrating that the COR theory [24,25] is an adequate explanatory theory when subjective well-being is a prominent mediator. In theorizing forgiveness climate as one way to halt the negative effect of workplace incivility on job satisfaction, our findings in support of forgiveness climate as a moderator in the workplace incivility and job satisfaction. Prior studies examined the moderating role of forgiveness climate between psychological contract breach and emotional exhaustion [23]. Our study is the first that explored the conditional effect of forgiveness climate in workplace incivility and job satisfaction association. Our research also extends workplace incivility research by examining its negative effect [33,34] on the employee’s subjective well-being. Prior studies have overlooked this relationship. The association was in line with earlier investigations investigating health-related consequences across sources of incivility [8,9,10,36,37]. Our study validated the findings of prior studies [38,39] that indicated that workplace incivility is associated with decreased job satisfaction in the health sector. Our study findings also reveal important points for minimizing the harmful impact of workplace incivility on job satisfaction and subjective well-being.

Our study found that incivility in the workplace has several negative effects [30,31], including decreased job satisfaction and subjective well-being. As a result, it is in the best interests of management to reduce the likelihood of it happening. Hospital management must be aware of these ramifications and understand that incivility might undermine the hospital’s high sustainability if it is not adequately addressed [36]. Management will benefit from this research in many ways. Employee personality qualities should be included in human resource management policies and practices, such as employee selection, training, promotion, and development, to lessen uncivil behavior. Management can reduce the incivility by supporting nurses and doctors, when they perceived the support from the organization [70,77]; they positively evaluate their life [49] and also feel satisfaction [58]. However, it is vital to avoid the undesirable consequences of workplace incivility. It is also necessary to resolve and minimize workplace incivility from occurring in the workplace [11]. An increasing body of evidence demonstrates that workplace intervention for reducing incivility and fostering civility can be effective [78]. Civility, respect, and engagement in the workforce (CREW) intervention, for example, encourages pleasant and respectful workplace relationships [48] by concentrating on individual behaviors in the context of a group, while also emphasizing provisions that assure management commitment [41].

### 5.2. Study Limitations and Future Scholarly Work

There are a few limitations in this research that are worthy of note. First, the data’s self-reported nature may be restrictive in terms of social desirability bias. Because of the data collected from hospitals, the findings’ generalizability might well be limited. A larger sample size, broader coverage, and an experimental data-gathering research approach could help us understand more. The strength of the relationships may have been inflated or deflated by cultural factors. Thus, future research might replicate the existing model in a Western society where everybody’s right to freedom of expression is respected. We measured the main variables at two different times, but our study still did not have a robust longitudinal research design since we have not measured changing effect of the study variables over time in the study model. That prohibits us from looking at the dynamic changes in the variables. We cannot say unequivocally that workplace incivility causes job satisfaction through subjective well-being, which may restrict our conclusions about the causal direction of the paths studied in the research model. In this study, we checked the moderating role of forgiveness climate in the workplace incivility and job satisfaction relationship. Thus, future studies may examine the direct impact of forgiveness climate on the job satisfaction. Moreover, future studies should consider job satisfaction as a mediator in the incivility and well-being relationship.

## 6. Conclusions

Our study investigated the indirect influence of workplace incivility on job satisfaction via subjective well-being, and also the conditional impacts of forgiveness climate. Our hypotheses received a lot of support: workplace incivility was associated with job satisfaction and subjective well-being. Nurses and doctors with a higher forgiveness climate felt more job satisfaction even amid workplace incivility. This research confirms the significance of paying attention to working settings and supporting good working situations to improve and maintain job satisfaction and well-being. This study adds to our understanding of how and why workplace incivility is associated with employee job satisfaction. It also explained the role of the forgiveness atmosphere in this process. Our findings have several practical implications and recommendations for activities and policies intended to reduce workplace incivility while also enhancing employee job satisfaction and subjective well-being.

## Figures and Tables

**Table 1:** Results of CFA.

Fit Indices	Hypothesized Model	Single Factor
CMIN (chi-square)	244.21	2988.83
DF (Degree of freedom)	129	135
CMIN/DF (Relative chi-square)	1.89	22.13
IFI (Incremental fit index)	0.97	0.44
NFI (Normed fit index)	0.95	0.43
CFI (Comparative fit index)	0.97	0.44
RMSEA (root mean square error of approximation)	0.03	0.17
TLI (Tucker-Lewis fit index)	0.97	0.37

**Table 2:** Construct Validity.

	Convergent Validity		Discriminant Validity			
Variables	CR	AVE	1	2	3	4
Job satisfaction	0.76	0.52	**0.72**			
Workplace incivility	0.89	0.54	−0.21	**0.73**		
Subjective well-being	0.86	0.55	0.32	−0.15	**0.74**	
Forgiveness climate	0.83	0.62	0.44	−0.13	0.18	**0.79**

Note: Values in bold in the diagonal represent the squared root estimate of AVE; Composite reliability = CR; average variance extracted = AVE.

**Table 3:** Construct Reliability and Correlation Coefficient.

Variables	α	WPI	SWB	JS	FC
Workplace incivility	0.89	-			
Subjective well-being	0.85	−0.13 **	-		
Job satisfaction	0.76	−0.17 **	0.26 **	-	
Forgiveness climate	0.83	−0.11 **	0.16 **	0.35 **	-

N (672), ** *p* < 0.01.

**Table 4:** Regression Results.

Direct Effect						
Hypotheses 1,2,3	β	SE	LLCI	ULCI	*p*	
H1: Workplace incivility-> Job Satisfaction	−0.46	0.10	−0.656	−0.284	0.000	
H2: Workplace incivility-> subjective well-being	−0.11	0.03	−0.181	−0.050	0.001	
H3: Subjective well-being-> Job Satisfaction	0.14	0.03	0.084	0.198	0.000	
**Indirect Effect**						
Hypothesis 4	β	SE	LLCI	ULCI	*p*	Z
Workplace incivility-> subjective well-being -> Job Satisfaction	−0.016	0.01	−0.031	−0.006	0.005	−2.777
**Interaction Effect**						
Hypothesis 5	β	SE	LLCI	ULCI	*p*	
Int_1 WPI × FC	0.10	0.03	0.056	0.152	0.000	

## Data Availability

The dataset generated and analyzed in the current study are available from the corresponding author upon reasonable request.
